# Viral hepatitis B and C in HIV-exposed South African infants

**DOI:** 10.1186/s12887-020-02479-x

**Published:** 2020-12-24

**Authors:** Cynthia Tamandjou Tchuem, Mark Fredric Cotton, Etienne Nel, Richard Tedder, Wolfgang Preiser, Avy Violari, Raziya Bobat, Laura Hovind, Lisa Aaron, Grace Montepiedra, Charles Mitchell, Monique Ingrid Andersson

**Affiliations:** 1 Division of Medical Virology, Department of Pathology, Faculty of Medicine and Health Sciences, Stellenbosch University, Cape Town, South Africa; 2 Department of Paediatrics & Child Health, Faculty of Medicine and Health Sciences, FAM-CRU, Stellenbosch University, Cape Town, South Africa; 3 Department of Paediatrics & Child Health, Faculty of Medicine and Health Sciences, Stellenbosch University, Cape Town, South Africa; 4 Blood Borne Viruses Unit, Virus Reference Department, Public Health England, London, UK; 5 Perinatal HIV Research Unit, University of the Witwatersrand, Johannesburg, South Africa; 6 Department of Paediatrics, Nelson R. Mandela School of Medicine, University of KwaZulu Natal, Durban, South Africa; 7 Frontier Science and Technology Research Foundation, New York, USA; 8 Center for Biostatistics in AIDS Research, Harvard T.H. Chan School of Public Health, Boston, Massachusetts USA; 9 Department of Paediatrics, School of Medicine, University of Miami Miller, Miami, USA; 10 Department of Microbiology and Infectious Diseases, Oxford University Hospitals NHS Foundation Trust, Oxford, UK

**Keywords:** Hepatitis B virus, HIV, Hepatitis C virus, Infants, South Africa

## Abstract

**Background:**

Whilst much attention is given to eliminating HIV mother-to-child transmission (MTCT), little has been done to ensure the same for hepatitis B virus (HBV) transmission. The introduction of HBV immunization at six weeks of age has reduced HBV horizontal transmission in South Africa. However, in order to eliminate HBV MTCT, further interventions are needed. The risk of hepatitis C virus (HCV) MTCT in HIV-infected (HIV+) African women is not yet well described. This study aimed to determine the rate of HBV and HCV vertical transmission in HIV-exposed infants in South Africa.

**Methods:**

Serum samples from infants enrolled in an isoniazid prevention study (P1041) were screened for HBV and HCV serology markers; screening was performed on samples collected at approximately 60 weeks of age of the infants. HBV DNA was quantified in HBsAg positive samples and HBV strains characterized through gene sequencing. All HCV antibody samples with inconclusive results underwent molecular testing.

**Results:**

Three of 821 infants were positive for both HBsAg and HBV DNA. All HBV strains belonged to HBV sub-genotype A1. The *rt*M204I mutation associated with lamivudine resistance was identified in one infant, a second infant harboured the double A1762T/G1764A BCP mutation. Phylogenetic analysis showed clustering between mother and infant viral genomic sequences. Twenty-one of 821 HIV-exposed infants tested had inconclusive HCV antibody results, none were HCV PCR positive.

**Conclusions:**

This study suggests that HBV vertical transmission is likely to be occurring in HIV-exposed infants in South Africa.. A more robust strategy of HBV prevention, including birth dose vaccination, is required to eradicate HBV MTCT. HCV infection was not detected.

## Background

Persistent viral hepatitis is a major public health problem and a major cause of infectious disease mortality, similar to HIV, malaria and tuberculosis [1]. Globally, hepatitis B (HBV) and hepatitis C (HCV) viruses affect an estimated 257 million and 71 million individuals, respectively. Sub-Saharan Africa (SSA), where the prevalence of chronic HBV infection is estimated at over 8%, and the Western Pacific region bear the greatest burden of HBV whilst HCV prevalence is highest in North Africa [2, 3].

Early regional studies in SSA described high HBV surface antigen (HBsAg) prevalence rates in children older than one year but low rates in infancy, implicating horizontal transmission at or after the neonatal period as the major route of transmission [4, 5]. These data informed the current policy of routine infant immunisation commencing at six weeks of age in many sub-Saharan African countries. Although this strategy led to a decrease in HBV infection in children [6], infants are still infected during the perinatal period [7]. HBV acquired during childhood carries a high risk for persistent infection and consequent severe liver disease. World Health Organization (WHO) guidelines recommend that the first dose of HBV vaccine be given shortly after delivery [8]; this is especially important in highly endemic regions such as SSA and the Western Pacific, where HBV mother-to-child transmission (MTCT) and early childhood transmission predominate. While implementation of this practice in the Western Pacific region has reduced both HBV MTCT and early childhood infections [9], HBV MTCT remains a concern in SSA [7]. Although current data suggest that immunisation starting at six weeks of age has reduced the prevalence of persistent HBV infection [6], the impact of HIV on HBV mother-to-child transmission (MTCT) in SSA is not well described. Moreover, little is known about the prevalence of persistent viral hepatitis in HIV-infected (HIV+) children.

About 1.96 million of 36.7 million HIV + individuals (~ 5.3%) in SSA are co-infected with HBV [10]. Of concern is the high prevalence of HBV e-antigen (HBeAg) in HIV + pregnant women [11]. In a recent Malawian study, 38.2% of 102 HIV/HBV co-infected women of childbearing age were HBeAg positive [12]. HBeAg prevalence rates of 18.9% (10/53) and 37.5% (6/16) have been reported in South Africa [13, 14]. HBeAg positivity is associated with higher HBV viral loads and increased risk of HBV MTCT [15]. In pregnancy, HBeAg positivity carries a 90% risk of HBV MTCT compared to 25% for HBeAg negative women [16]. Prevention of HIV MTCT (PMTCT) guidelines recommend routine HIV testing for all pregnant women with unknown HIV status. However, as HBV antenatal screening is not routinely performed in many parts of SSA, the extent of perinatal HBV transmission in the HIV era remains under-investigated. Whilst there is some epidemiological data in blood donors and antenatal cohorts, there are few reports in children [17].

HCV prevalence is considered low in SSA [18], but little is known about HCV in pregnancy and in children, in particular in those who are HIV co-infected. This study aimed to establish the prevalence of HBV and HCV infection in infants of HIV + mothers in South Africa.

## Methods

### Study design

This was a retrospective study using residual samples from the P1041 trial through the International Maternal Paediatric Adolescent AIDS Clinical Trial (IMPAACT) Group. P1041 was a multi-centre, Phase II-III randomized, double blind, placebo-controlled trial to determine the efficacy of isoniazid in preventing tuberculosis (TB) disease and latent TB infection among infants perinatally exposed to HIV [19]. From December 2004 to June 2008, 1354 infants born to HIV + mothers in South African sites including Cape Town, Durban, and Johannesburg, and Gaborone in Botswana were enrolled in the P1041 study. Among these 1354 infants, 548 were HIV-infected and 806 were HIV-uninfected. Collection of data and blood samples occurred at three time points: at study entry, which corresponded to approximately 12 weeks of age (“Week 0” on study), at ~ 24 weeks of age (“Week 12” on study) and ~ 60 weeks of age (“Week 48” on study). The HBV infection status of the mothers was unknown.

Available plasma and serum samples from “Week 48” from the South African sites were screened for active HBV and HCV infections. Samples collected at earlier time points (i.e. “Week 0” and “Week 12”) from infants found HBsAg positive at “Week 48” were tested for active HBV infection, and attempts were made to trace all HBsAg positive infants and their mothers for serological and molecular follow-up testing. Universal HBV immunisation at 6, 10 and 14 weeks of age was introduced in South Africa in 1995, preceding the P1041 trial by nine years.

### Serological screening

Given the limited sample volumes available, serological testing was done at 1:10 dilution in normal human plasma, on the Abbott ARCHITECT i2000SR automated platform (Abbott Diagnostics, Delkenheim, Germany), following previous validation (data not shown).

Samples were tested for HBsAg, anti-HBs, anti-HBc (total) and anti-HCV using the ARCHITECT i2000SR system, according to manufacturer’s instructions. HBsAg positive samples were tested for HBeAg and anti-HBe using the ARCHITECT HBeAg and anti-HBe chemiluminescent immunoassays. Anti-HBs titre ≥ 10 IU/mL is the accepted threshold for protective immunity against HBV. Samples with anti-HBs titres below 1 IU/ml were recorded anti-HBs negative. Samples with anti-HBs levels falling between 10 IU/ml and 100 IU/ml, indicator low level of immune protection against HBV, were also recorded.

### Quantification of HBV DNA

Viral DNA was extracted from 200 µl of serum samples using the QIAamp® MinElute® Virus Spin kit (QIAGEN, Hilden, Germany). Extracted HBV DNA was amplified and quantified, using a probe-based real-time PCR assay on the RotorGene™ 6000 (Corbett Life Sciences, Australia), at the following cycling conditions: initial denaturation for 15 minutes at 95 °C followed by 45 cycles of 95 °C for 15 seconds and 60 °C for 60 seconds [20]. Sequences of the primers and probes used in the assay were as described by Garson et al. (2005) [20].

### Quantification of HCV RNA

Viral nucleic acid extraction and quantification were performed using the COBAS® AmpliPrep/COBAS® TaqMan® HCV Test (Roche Molecular Diagnostics, Almere, Netherlands), according to manufacturer’s instructions.

### HBV DNA sequencing

DNA positive samples underwent sequencing of the *polymerase/surface* and *core* genomic regions [21, 22]. Genomic sequences were submitted to the National Center for Biotechnology Information HBV Genotyping tool (http://hivdb.stanford.edu/HBV/HBVseq/development/HBVseq.html) and to Geno2Pheno (http://hbv.geno2pheno.org/index.php) for HBV genotyping and to detect immune escape and drug resistance mutants, respectively.

### Phylogenetic analysis

The relationship between maternal and children viral sequences was inferred using a Maximum Likelihood phylogenetic tree constructed using a bootstrap of 1000 replicates in MEGA 7 [23]. Evolutionary distances between the query sequences were calculated using the General Time Reversible model [24].

## Results

The study population comprised of 821 infants from Cape Town, Durban, and Johannesburg with plasma samples available from “Week 48”. Baseline demographics and HIV clinical characteristics are outlined in Table 1.

**Table 1:** Participant demographics and HIV characteristics of study population^a^

Characteristics	Study population ***N***= 821 (%)
**Age (in weeks)**	
Mean	62
Min - Max	49 - 64
**Sex (%)**	
Male	398 (48%)
Female	423 (52%)
**HBV vaccination**	
Yes	129 (16%)
Unknown	692 (84%)
**HIV infection**^**a**^	
Yes	315 (38%)
No	506 (62%)
**On ART at sample collection**^**b**^	
Yes	259 (82%)
No	56 (18%)

^a^Assessed as of date of sample collection at Study “Week 48”; ^b^ART: combination Antiretroviral Therapy, defined as being on at least 3 drugs in at least 2 classes

### Active HBV infection screening

Among screened samples, three samples were positive for HBsAg and HBV DNA (3/821, 0.4%), of which two were from HIV + infants and the other from an HIV exposed uninfected infant, one each from Cape Town, Johannesburg, and Durban. Five samples were anti-HBc (total) reactive (5/821, 0.6%); of these, three were also HBsAg positive while the other two were HBsAg negative. Insufficient sample volume prevented HBeAg and anti-HBe testing (Table 2). Sequencing of the HBV *polymerase/surface* and *core* genomic regions showed sub-genotype A1 strains. Sequence analysis revealed the M204I mutation in the reverse transcriptase domain of the *polymerase* gene in the Durban sample while the Cape Town sample harboured the double A1762T/G1764A BCP mutation in the *core* gene. No HBV mutation was found in the Johannesburg sample.

**Table 2:** Serological and molecular results of HBV-infected children at screening and follow-up

Patient	Location	HBV vaccination	HIV Status	Screening		Prospective follow-up			
				HBsAg	HBV DNA log 10 (IU/ml)	HBsAg	HBeAg	Anti-HBe	HBV DNA (IU/ml)
**C1**^**a**^	CPT	Yes	+	+	8.93	+	-	+	UD
**C2**	Durban	No record	+	+	5.17	ND	ND	ND	ND
**C3**	JHB	No record	-	+	8.45	ND	ND	ND	ND

^a^Therapy: D4T, 3TC & KLT (at follow-up); *Anti-HBe* antibody to hepatitis B e antigen, *C* Child, *CPT* Cape Town, *IU/ml* International Units per milliliter, *HBeAg* hepatitis B e antigen, *HBsAg* hepatitis B surface antigen, *JHB* Johannesburg, *−* Negative, *+* Positive, *ND* Not done, *UD* Undetectable

### Retrospective serological and molecular testing

One HBsAg positive infant (Cape Town) had been immunised against HBV, while there were no records of HBV immunisation for the Durban and Johannesburg HBsAg positive infants. Samples from earlier time points were retrieved for the Durban and Cape Town infants and were screened for active HBV infection. Sample volume was sufficient for serological and molecular screening for the “Week 0” Cape Town sample and molecular testing only for the “Week 0” and “Week 24” Durban samples. All three samples were HBV DNA positive. The “Week 0” Cape Town sample showed a similar serological profile to the “Week 48” sample from the same child and also had the double A1762T/G1764A BCP mutation. Insufficient sample volume precluded testing the Johannesburg infant.

### Follow-up

Two HBsAg positive infants (Durban and Johannesburg) could not be traced, but the HBsAg positive infant from Cape Town was traced and reviewed seven years after the “Week 48” screening assessment, at 8 years of age. This child was HIV + and receiving stavudine (D4T), lamivudine (3TC) and lopinavir/ritonavir (LPV/r) while the mother was on 3TC, tenofovir (TDF) and LPV/r.

### Follow-up serological and molecular testing

**Fig. 1:**
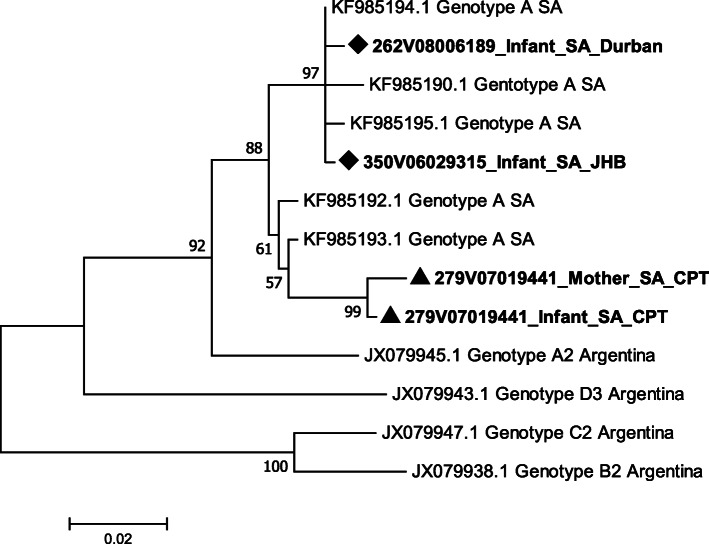
Phylogenetic tree of HBV-infected infants and Cape Town mother based on the HBV *core *region. Sequences with accession numbers starting with JX and KF were downloaded from GenBank. The evolutionary history was inferred by using the Maximum Likelihood method based on the General Time Reversible model with 1000 bootstrap replicates. A discrete Gamma distribution [25] was used to model evolutionary rate differences among sites

Samples from both mother and child were HBsAg positive, HBeAg negative/anti-HBe positive at follow up. While the child sample had an undetectable HBV viral load, low level HBV DNA was detected in the maternal sample (HBV viral load = 19 IU/ml). Owing to the low maternal viral load, only core sequencing was successful. A comparison of the maternal and the “Week 48” infant HBV core sequences showed similarity in protein/nucleotide sequences (99% homology). Both sequences harboured the double A1762T/G1764A BCP mutation. Phylogenetic analysis showed close clustering between the maternal and child sequences (Fig. 1).

### HBV immunity screening

HBV immunity, demonstrated by the presence of anti-HBs only, was assessed in all 821 samples. Seroprotective levels of anti-HBs ≥ 10 IU/ml were found in 745/821 samples (90.7%); of which 277 (37.2%) had anti-HBs levels < 100 IU/ml. The remaining 76 (9.3%) samples had anti-HBs levels < 10 IU/ml; of which 14 samples (1.7%) were anti-HBs negative (< 1 IU/ml). Among the five anti-HBc (total) reactive samples, the two HBsAg negative samples had anti-HBs levels above 100 IU/ml while the three HBsAg positive samples had anti-HBs levels below 10 IU/ml.

### HCV infection screening

All 813 “Week 48” samples tested for anti-HCV were negative. However, 21 samples showed S/CO values > 0.10; this was thought to be due to non-specific reactivity. In order to address the possibility that antibody response may have been impaired and low, HCV RNA testing was undertaken on 16/21 samples with sufficient volume available, with negative results.

## Discussion

In this cohort of infants born to HIV + mothers, three infant samples were HBsAg positive at “Week 48”, giving a 0.4% prevalence of active infection (3/821). Testing for anti-HBc (total) showed that a further two infant samples (2/821, 0.2%) showed the presence of anti-HBc (total) levels greater than the assay’s cut-off (> 1 S/CO), suggesting past infection to HBV. On repeat testing the Cape Town case revealed persistent HBV infection with a strain closely related to the maternal HB virus. This was despite infant HBV immunisation. The other two cases could not be traced.

In South Africa, HBV immunisation from six weeks of age was implemented in the mid-1990s as HBV transmission was thought to be predominantly horizontal [4, 26, 27]. This intervention led to a decrease in HBsAg prevalence rate from approximately 10% [28] to between 0% and 2.7% [29–33]. A pre- versus post-HBV immunization analysis of 1206 children aged 1 to 25 years in South Africa showed an HBsAg prevalence rate declining from 4.2–1.4%, and prevalence of HBV immunity increasing from 13–57% [6]. Although HBV immunisation was documented in only 16% (129/821) of our cohort, 90.6% (745/821) had protective anti-HBs levels, suggesting poor documentation rather than deficiencies in immunisation coverage.

Although the prevalence of HBV infection in childhood has declined, HBV perinatal transmission remains a concern. Delay in seroconversion to anti-HBe, reactivation of HBe antigenemia and sero-reversion to HBsAg positivity, indicative of increased HBV replication through HIV-induced immunosuppression, are well described in HIV + patients [34, 35]. High HBsAg and HBeAg prevalences with high HBV replication activity have been described in HIV + South African women [13]. Furthermore, a study from Soweto described four cases of perinatal transmission from 14 HIV + mothers. Three of the four infants were born to HBeAg positive mothers with HBV viral load of 1.995E + 8 IU/ml, 6.309 IU/ml and 100 IU/ml HBV DNA [36].

The present study showed a HBsAg prevalence (0.4%, 3/820) similar to that in 1000 HIV-exposed infants from the Western Cape’s PMTCT program [37]. In both studies, HBV immunisation starting at six weeks of age failed to prevent all HBV MTCT.

It is well established that HBV immunisation at birth alone is effective in preventing HBV perinatal transmission from some HBV-infected women [38–40] but is only partially protective for infants born to mothers with high HBV viral loads [41]. Giving hepatitis B immunoglobulin (HBIG) together with HBV birth vaccine, active/passive immunisation further reduces the risk of HBV MTCT [38, 42, 43], but is costly and not logistically possible in many resource-limited settings. Treating women in the third trimester of pregnancy with antiviral drugs such as lamivudine [44], tenofovir [45] or telbivudine [46] is effective in reducing HBV viral load and thus reducing the risk of transmission. Tenofovir and emtricitabine (or lamivudine) have been part of first-line antenatal antiretroviral therapy (ART) in SSA since 2010, so HIV-HBV co-infected pregnant women would have received anti-HBV therapy. However, HBV mono-infected pregnant women remain unidentified and untreated, leaving their infants at risk of acquiring HBV from their mothers.

The other interesting aspect of these results is to consider the 2/5 anti-HBc (total) reactive samples, which may indicate exposure to HBV infection, but may also reflect residual maternal antibody.

No HCV infection was identified in this study. HCV prevalence is considered low in SSA (< 1%) [18]. However, there is little published data from paediatric cohorts in this region. Further work is required to confirm the findings of this study.

While this study adds to our understanding of HBV epidemiology in South African infants, there are limitations in the interpretation of these results. The absence of ART data on mothers prevented assessing the effect of maternal ART on the HBV infection risk to infants. However, during the study period, TDF and emtricitabine (FTC) were unavailable and only HIV + mothers with a CD4 + T-cell count below 200 per mm^3^ were eligible for lamivudine-containing ART, which would have reduced the risk of HBV transmission. Currently in South Africa all HIV + women have access to ART containing TDF and lamivudine or FTC. In the absence of HBV screening in pregnancy, the risk of replacing tenofovir to a second- or third-line ART may not be appreciated. Maternal HBV status would have allowed calculation of a definitive risk of perinatal transmission but was unavailable. Limited immunisation records compromised the interpretation of anti-HBs levels. Finally, as the samples were collected more than seven years ago from three different locations, loss to follow-up prevented further assessment of two of the HBsAg positive cases.

## Conclusions

This study shows evidence of HBV infections in infants born to HIV + mothers. No HCV infection was detected in this large cohort of HIV-exposed infants in South Africa. In order to eliminate HBV MTCT, a combination of strategies including HBV screening, together with maternal antiviral therapy and most importantly HBV immunisation at the time of birth will be required. These strategies, although well described, require further SSA data to assess the cost effectiveness ultimately to eliminate this route of HBV infection.

## Data Availability

The datasets during and/or analysed during the current study are available from the corresponding author on reasonable request.

## Abbreviations

3TC: Lamivudine
Anti-HBc (total): Total antibodies against hepatitis B core antigen
Anti-HBe: Antibodies against hepatitis B e antigen
Anti-HBs: Antibodies against hepatitis B surface antigen
Anti-HCV: Antibodies against hepatitis C virus
ART: Antiretroviral therapy
BCP: Basal Core Promoter
CD4 + T-cell: Type CD4 + T cell
D4T: Stavudine
DNA: Deoxyribonucleic acid
FTC: Emtricitabine
HBeAg: Hepatitis B e antigen
HBsAg: Hepatitis B surface antigen
HBV: Hepatitis B infection
HCV: Hepatitis C virus
HIV: Human Immunodeficiency Virus
LPV/r: Lopinavir/ritonavir
MTCT: Mother-to-child transmission
PMTCT: Prevention of mother-to-child transmission
RNA: Ribonucleic acid
SSA: Sub-Sahara Africa
TB: Tuberculosis
TDF: Tenofovir Disoproxil Fumarate
VL: Viral load
WHO: World Health Organization
